# Employer support for health and social care registered professionals, their patients and service users involved in regulatory fitness to practise regulatory proceedings

**DOI:** 10.1186/s12913-024-11646-0

**Published:** 2024-10-22

**Authors:** Louise M Wallace, Mari Greenfield

**Affiliations:** 1 Faculty of Wellbeing, Education and Language Studies, Open University, Walton Hall, Milton Keynes, UK; 2 The Open University, Walton Hall, Milton Keynes, MK7 6AA UK

**Keywords:** Regulation, Registrant, Fitness to practise

## Abstract

**Background:**

Health and social care employees may be involved in professional regulatory proceedings because their alleged behaviour raises health or conduct concerns. Employees, patients or service users may also be involved in a regulatory tribunal as witnesses. This study is about the role of employers in supporting them in this process.

**Methods:**

Taking an organisational support perspective, we interviewed 25 senior employees in health and social care organisations in the UK who are responsible for managing the employer’s role in the proceedings. Template analysis was used to analyse the transcripts.

**Results:**

Support for patients was limited to that offered during an employer’s complaints process, and only one employer gave some support during the regulatory process if the patient or service user initiated regulatory proceedings. Support for employees in tribunals was little different to that offered during an initial investigation by the employer. However, where additional support for being involved in a regulatory tribunal was offered to employees, it most often came from the senior employees’ own experience of the stressfulness of being a witness in these proceedings. Employers were not aware of training resources that would better support their employees to engage with professional regulatory proceedings.

**Conclusions:**

Employers offer limited support to employees who are involved in professional regulatory proceedings, and the support offered may depend more on senior employee’s individual experience of involvement in regulatory proceedings to exercise this discretion. Patients, service users and other public witnesses receive almost no support from health and social care employers during professional regulatory proceedings.

**Supplementary Information:**

The online version contains supplementary material available at 10.1186/s12913-024-11646-0.

## Introduction

In the United Kingdom (UK), professionals must be qualified to register with their regulatory body and thereby be permitted to practise. Registering also entails adhering to the profession’s standards, as well as acting within the law. If practice is called into question, registrants can be referred to their regulator for investigation under fitness to practise proceedings (FtP). The UK has 9 statutory health regulators, and one regulator in each of the four UK countries for social work, (of which 3 include a wider social care workforce) [1]. There are also voluntary accredited registers, but these were not included in the study as numbers of FtP proceedings are small.

Professional regulators have no powers of inspection; they rely on people or organisations “notifying”, “referring” or raising a concern about a registrant. Concerns may come to regulators from the public, directly from colleagues, or organisations such as the police, or an employer, usually following their own disciplinary investigation. The emotional and health consequences of the employers’ complaints investigation on registrants is well documented, and can be associated with adverse health outcomes [2]. A smaller but growing body of research shows the regulatory processes have serious adverse consequences for the health of some registrants [3, 4], including for some doctors, suicide [5, 6]. 

Registrants may be involved in regulatory proceedings as witnesses, providing evidence of what they observed or themselves experienced in relation to a registrant. It has been known for more than two decades that involvement in a serious adverse event can have lasting emotional and health consequences for the professionals involved. The contested term “victim” is used to apply to the person who was in some way harmed by the adverse event. The term ‘second victim’ (developed from war veteran literature) refers to social or healthcare professionals who experience emotional distress following an adverse event, whether they were directly involved or an observer [7, 8]. This distress has been shown to be like that of the patient—the ‘first victim’. The extent of the enduring distress professionals suffer is related to the amount, or lack of, organisational support received [7, 9]. Previous research does not, however, identify additional impacts of subsequent professional regulatory processes. Also, these studies do not differentiate between the “actor” and “observer,” i.e., employees who witnesses the event but whose conduct is not under investigation, a distinction investigated in our study.

The public make a substantial proportion of concerns to UK regulators. For example, 70% of new complaints in the 40 months since the inception of Social Work England were from the public, (personal communication, Social Work England). In the most recent year 74.7% of concerns about doctors raised with the General Medical Council and 38% for nurses and midwives to the Nursing and Midwifery Council (NMC)were from the public [10, 11]. Research commissioned by regulators shows there is scant public knowledge about healthcare professional regulation, and perhaps even less awareness for social work [12]. Studies of healthcare complaints show under-representation of vulnerable groups [13], meaning complainants may be an unusual, perhaps more economically and socially resourced sub-sample. Public witnesses’ needs for support by the regulators is acknowledged on some regulators’ websites [14]. 

There is a small body of research commissioned by UK regulators examining registrants’ experiences [15], employers, and the wider public with varied involvement in regulatory proceedings [16, 17], and a large study of health professionals and patients and their families involved regulatory proceedings [18]. This type of research investigates how processes can be improved, rather than registrants’ or the public’s support needs.

If a registrant has been referred to their regulator, their case may involve other employees who give witness evidence. It may also involve members of the public, either as the referrer or as a witness. The role of the employer in supporting employees as witnesses to allegations when giving evidence in a tribunal, or as the referred registrant in the UK has not been investigated. Studies of adverse incidents show perceived organisational support by colleagues and managers enhances belief that the organisation values a person’s contribution and care for their well-being as an employee. It can reduce the traumatic consequences of work stressors [19], and reciprocally can increase the contribution of workers’ extra role performance such as informal support for colleagues [20]. 

There is no research we are aware of on the role of health and care organisations in supporting patients/service users and their families through regulatory proceedings, despite harrowing personal accounts of the impact of FtP processes on those harmed [21, 22]. Organisations may not be aware of the regulatory concern until it is investigated by the regulator when it is raised by a member of the public. This study therefore also sought to establish if and how employers provided support to patients and service users involved in FtP proceedings.

### Research aims

This study sought to establish the support offered by employers to employees and to patients/service users involved in FtP proceedings. As the ability to offer support is based on an understanding of the needs that support would meet the study also sought to establish what employers understood about the needs of employees and patients/service user involved in FtP proceedings. Employees includes referred registrants and those who are witnesses to allegations against a colleague, whose behaviour they may have observed and/or who may have directly harmed them.

Research question: How do employers understand their responsibilities to support employee and public witnesses in FtP proceedings?

## Methods

### Overall approach

Semi-structured interviews were carried out with 25 senior managers within UK social and healthcare organisations employing registrants, using an interview guide developed for this project (see Supplementary Materials for interview guide). Remote interviews facilitated inclusion of participants from all four UK countries, and were audio-recorded and transcribed before analysis. Ethical approval was granted by The Open University (OU HREC No. HREC/4058/Wallace).

### Sampling, recruitment and consent

#### Sampling

Included participants had formal responsibility for referring registrants to health or care regulators in a UK organisation. Purposive sampling was used across a range of regulators and sectors, including non-public sector and corporate business, social or healthcare employers. Those included were sampled in categories to obtain representation across; (1) sectors (health, social care) (2), organisation types (public, private for profit or non-profit), and (3) UK countries.

#### Recruitment

A multi-strand approach to recruitment was used, initially utilising employer networks of regulators from organisations contacted for another project (NIHR 129491), and health and social care panel members from an NIHR funding committee. Included organisations provided health and/or care services in the UK involving staff registered with one or more of the UK statutory regulators. Snowball methodology was then employed; interviewees suggested other potential participants with different professional remits within their own organisation, or counterpart colleagues in similar organisations [23]. 

Participants either gave written consent via an online link, or verbal consent at the beginning of their interview.

### Analysis

Quantitative data derived from interview transcripts was reported in a descriptive form [24]. Qualitative data was analysed using template analysis – a codebook style of thematic analysis widely used in organisational research and within the social and healthcare sectors [24, 25]. 

Categorical data such as employee numbers were tabulated. Text was analysed to ascertain types of support offered and how this differed dependent on role. Preliminary codes were devised, then clustered into themes and subthemes which became the coding template. Definitions of themes and relationships between them were continually refined throughout the analytic process [26], and the coding template was updated after each transcript had been analysed. Once all transcripts were processed a final template was produced and then re-applied to each transcript [26]. 

## Results

### Organisational type, regulator and registrant numbers

There was wide variability in the number of registrants interviewees estimated they were responsible for (25 − 10,000), and hence the number of new FtP cases they dealt with annually (1–65). Quantitative data relating to interviewees’ organisational type, regulators, registrants and FtP case numbers are shown in Table 1. Employers spanned large NHS and Local Authorities along with a mix of private health including corporate pharmacy businesses, and social care providers of varying sizes, which worked with ten regulators.

**Table 1:** Interviewees’ organisational background and FtP responsibilities

Organisational type and sector	Number of interviewees		Regulators worked with	Registrant types	Estimated number of registrants interviewee was responsible for (range)	Estimated number of FtP cases (range) interviewee responsible for in past year.
NHS (*n* = 12)	NHS Board	3	Health Care Professions Council (8) Nursing & Midwifery Council (8) General Medical Council (2) Social Work England (2) General Pharmaceutical Council (1), General Optical Council (1) General Osteopathic Council (1)	Doctors Psychologists Dentists Healthcare scientists Optometrists Pharmacists Physician associates Allied health professionals Osteopaths	130–10,000	1–30
	NHS General hospital and community services Trust	3				
	Mental health and community services Trust	6				
Private health and/or social care provider (*n* = 6)	Private mental health/learning disability care	5	Health Care Professions Council (6) Nursing & Midwifery Council (4) Social Care Wales (3) Scottish Social Services Council (2) Social Work England (1)	Social workers Nurses Allied health professionals Social care staff	75–3,000	1–65
	Private residential care for older people	5				
	Private residential care for children	3				
Corporate pharmacy business (*n* = 3)	3		General Pharmaceutical Council (3) Pharmaceutical Council of Northern Ireland (1) Nursing & Midwifery Council (1) General Medical Council (1)	Pharmacists, nurses, doctors	750–7,000	6–12
Public sector social care = 4)	4		Social Work England (3) Health Care Professions Council (1)	Social workers Allied health professionals	25–400	1–3

### Organisational support themes

Three themes were produced from the data – Registrants, Colleague Witnesses, and Public Witnesses each with sub-themes. These are detailed in Table 2, alongside illustrative quotes and referred to below in discussion of the themes.

**Table 2:** Thematic map

Theme	Sub-theme	Illustrative quotes
Registrants	Personal experiences of FtP	‘So even if I had to look for a job, I couldn’t, I have to wait, so it’s those kind of things. And I’ve seen it in our registrants as well and it is really frustrating.’ (Interview 7) ‘I was the investigating manager, so I provided evidence at that case’ (Interview 11)
	Appreciation of referred registrants’ needs	‘The mental health of people involved in these investigations… must be impacted. If you have something like this hanging over your head, whether you’re a witness or a registrant, and this is over your head for 12-18-36 months, that is really going to affect you in lots of ways.’ (Interview 15) ‘It was only recently where she was given an outcome of the fitness to practise, and then the press got hold of it and we had to call her up to say, are you aware that this is in the papers? And she was destroyed. And went off sick, went to GP, had to get some medication. {…} I just feel for frontline support worker {…} for the fitness to practise team to take three years to make a decision and then for the press just to get a hold of it and then bring it all back up again. There must be a better way of dealing with things like this or, you know, a bit quicker.’ (Interview 25)
	Organisational support for referred registrants	‘So we provide occupational health and that’s entirely confidential. It doesn’t need to be a management referral. I would always make sure that any individual knows they can make a self-referral to occupational health. And within occupational health we have peer support and counselling support as well’ (Interview 2) ‘I’ve gone out personally to the house to see her when she was off sick to try and offer that support. So my management team here and my supervisors and that and are always on hand to kind of phone up do welfare checks as I said we’ve got the employee advisory programme system there as well in place for staff and we do a lot in mental well-being and everything as an organization so it’s something that’s, and it’s really important to us to try and support staff at that level.’ (Interview 25)
Colleague witnesses	Appreciation of colleague witnesses’ needs	‘The referral was made, I want to say late 2018, early 2019, then we kept being asked for more and more information, more and more details, then we got the pandemic, so the case was only heard in February… luckily all witnesses were still employed in the trust so we were able to kind of support.’ (Interview 6) ‘The [regulator] takes so long, often the incident you’re going to be a witness about might have happened three, four years ago, and often you cannot remember. And you can say I cannot remember, but they’ll keep pushing you. And a colleague, in the feedback, [the regulator reported] she wasn’t a very reliable witness. And the thing is they write that publicly afterwards… And you have no choice to go. If you’re a [registrant], you are expected to go.’ (Interview 9)
	Organisational support for colleague witnesses	‘We had a case where a couple of our health visitors had to go and be witnesses, so at our next conference we got them to do a bit of a session on this is what it’s been like to be a witness at a professional body hearing.’ (Interview 6) ‘There’s not something like you can phone a number and somebody will come to you or do anything like that. There is nothing like that in place at all. And I do think that’s again that’s something I would say we lack.’ (Interview 4)
	More appropriate processes	‘It might be two colleagues who worked on a team together, what’s the effect that’s potentially had on the team, are we going to build that again within the team? There will be alliances that have been formed as part of that.’ (Interview 12) ‘we’ve recently had one very vexatious registrant… that has referred I think probably about 12 people… he’s referred people, if they… just so happened to be a registrant and they’ve chaired a disciplinary process. I’ve personally formally complained to the [regulator] about some of their processes in relation to this particular case.’ (Interview 8).
Public witnesses	Lack of knowledge about public witnesses’ needs	‘So the [regulator] don’t necessarily tell us…they don’t tell us who’s referred an individual.’ (Interview 8) ‘The one that the… foster carer had made the complaint to the regulator, I was expecting them to have a being called because it was them who submitted the complaint. Whether they were or not, I don’t know, because I have never had feedback from [legal firm] or {regulator}about what happened to the case.’ (Interview 20)
	Response to public witnesses’ needs	‘I don’t know that we are necessarily very good at that, if I’m being honest. I think in some instances we might not even see that as being our role, if I’m being honest… we just assume, rightly or wrongly, that because… it’s not our process, it’s not our responsibility.’ (Interview 5) ‘I’ve never actually thought about it until you’ve asked that question because it actually would be very, it might restorative to that family if the organisation actually reached out and said can we help you in this situation?’ (Interview 3)

#### Registrants’ needs and support

##### Appreciation of referred registrants’ needs

All interviewees were acutely aware of the stress that FtP caused to registrants. They summarised the process for registrants as:

‘Either they’re going to lose their licence to practice, they’re going to have limitations on their practice, or they’re going to have to go through a process that gets to that end or doesn’t, but it’s still not a nice process.’ (Interview 6).

The length of time from referral to outcome was commented on by all interviewees and was seen as damaging to the registrant and frustrating to the organisation. The lack of information provided to the registrant or their employer when a referral had been made by someone other than the employer was seen as creating a barrier to the provision of organisational support. The registrant’s potential awareness of the referral and simultaneous lack of awareness of the details for an extended period was felt to be both emotionally harmful to them, and out of the control of the organisation to resolve. Interviewees also described the career consequences of lengthy proceedings, where referred registrants might not apply for or be considered for promotions whilst under investigation. Regardless of the outcome, the referral might have harmed the registrant’s reputation within the organisation:

‘If a person is suspended without prejudice, you can’t go and tell their team they’ve been suspended because there’s an allegation of bullying. So you say Rob’s not at work at the moment and he can’t make contact with anybody and everybody thinks what’s happened? That’s a very uncomfortable place… it can be difficult even when it’s resolved for the person to return.’ (Interview 2).

Many interviewees felt their organisations were not able to provide the practical support registrants might need in these circumstances.

##### Personal experiences

In addition to reporting the experiences of referred registrants they were responsible for, interviewees also had personal experience of FtP tribunals, either as a colleague witness (*n* = 8), or of being referred themselves (*n* = 3), and of sitting on the regulator’s panel (*n* = 1). Interviewees described how these experiences gave them insight into the difficulties faced by referred registrants and witnesses.

[Discussing personal experience of being a tribunal witness] ‘It was awful. And I have a number of colleagues who have been through the same. So much so that now if we have someone going to the [regulator] we send someone with them. We make sure that they’ve got a senior member of staff going with them because it’s, the way they were made to feel was appalling. And the way I was made to feel was really really awful, really awful.’ (Interview 9).

##### Organisational support for referred registrants

Within larger organisations, interviewees reported that structures existed to separate management roles between disciplinary investigations and support for referred registrants. The importance of having both roles, clearly divided, was stressed by interviewees, particularly in organisations with higher numbers of referrals. All interviewees named support available to referred registrants, regardless of how many referred registrants they had previously had responsibility for (see Table 3). Support was seen as essential for registrants’ health, retaining them within the profession, and even preventing suicide:

‘I’ve been seeing quite a few doctors dying by suicide and people think there’s a link with formal [regulator] process.’ (Interview 7).

Table 3 describes the support available to referred registrants, as well as to those we consider next, harmed and unharmed colleague witnesses. All described some support, but the sources varied widely, with some form of organisational wellbeing support such as occupational health, employee support services or the line manager’s support being the most often cited across all sectors and organisational types. There were a few instances of organisations providing colleague witnesses with preparation for giving evidence, or support from independent legal or mediation services.

Some organisations in all sectors also offered support to prepare for and/or accompany people to tribunals, with little difference by sector or public/corporate employer.

**Table 3:** Organisational support offered to registrants and colleague witnesses

**Organisational type and sector**	**Registrants**		**Colleague witnesses**	
			**Unharmed**	**Harm alleged**
	**Support mentioned**	**Number who mention**		
NHS (n=12)	Organisational wellbeing support	12	12	10
	Freedom to speak up guardian	1	1	1
	Booklets (explaining FtP processes)	-	1	-
	Discussion of hearing processes	1	4	2
	Person to accompany to hearing(s)	1	5	2
	Named link person/senior manager	4	3	-
	Line manager (as only support)	-	-	2
	Mediation and resolution service	-	-	2
Private health and/or social Care provider (n=6)	Organisational wellbeing support	6	6	4
	Person to accompany to hearing(s)	1	1	1
	Support to refer to Police	-	-	1
Corporate pharmacy business (n=3)	Organisational wellbeing support	3	3	2
	Person to accompany witness to hearing(s)	-	3	2
Public sector social care (n=4)	Organisational wellbeing support	4	3	4
	Person to accompany registrant to hearing(s)	-	1	1

#### Colleague witnesses (see Tables 2 and 3)

These are employees who may have been harmed by another employee (a colleague) and raised concerns about that person, or who were witnesses to a colleague’s misconduct with another person (staff or patient/service user) and called to give evidence.

##### Appreciation of colleague witnesses’ needs

Interviewees demonstrated a clear understanding of colleague witnesses’ needs, whether they were harmed by the registrant, or were an unharmed witness to the registrant’s behaviour.

The length of time between referral and outcome was identified as problematic for colleague witnesses. Alongside the extended emotional burden, difficulties included employees having left their organisation, or not being able to recall events. Employees having left the organisation, or having been employed via an agency, prevented employers from offering support. Interviewees contrasted these lengthy delays with the timescales given to colleague witnesses to provide evidence or statements, noting that these were frequently very short, which was seen as both unfair and an unreasonable pressure especially upon those in clinical roles.

‘The people who are providing these statements…are clinicians, they’re people who are looking after patients and expecting them to be able to respond so quickly just puts too much pressure, it risks the quality of what you get then and the accuracy of that… and that’s patient care you’re about to impact on and patient safety’. (Interview 6)

Interviewees discussed the difficulties they faced in offering effective support in a situation which they perceived as inherently unfair, and where their employees needs as clinicians were not recognised by the regulators’ legal services. Interviewees also recognised the inherent difficulties of being a witness in a colleague’s FtP tribunal, recognising the duty that the organisation had to manage these difficulties for staff.

All interviewees said registrants attending FtP tribunals as witnesses needed good quality information about what to expect. The materials provided by various regulators were described as insufficient, but few produced their own formal materials. Most interviewees felt that regulators should be responsible for the provision of information on their processes. Many interviewees said that registrants who were called to be FtP witnesses approached them for support rather than the regulator:

‘we’ve had lots of people reach out when they get contacted by the lawyers, that obviously stresses people quite considerably, so we’re here to support them as well’. (Interview 13)

##### Organisational support for colleague witnesses

To meet colleague witnesses’ need for information about FtP processes, interviewees described a range of provision. As Table 3 shows there was very little variation according to whether the colleague was harmed or a non-harmed witness to the registrant’s behaviour. Whilst all 25 interviewees described some provision of advice about preparing a statement (sometimes from the employers’ legal adviser), many relied upon witnesses themselves and their line managers to decide on appropriate well-being support.

‘We do try to have conversation with them around this is what you’re going to experience. This is what it will be like.’ (Interview 1).

Other interviewees provided support themselves, having conversations with employees about the processes and tribunal. Others described informational booklets their organisations provided. One organisation arranged for employees who had been tribunal witnesses to disseminate their experiences, and two interviewees said their organisations suggested colleague witnesses attend tribunals as members of the public prior to attending as a witness. Some organisations provided significant ongoing support for colleague witnesses, including accompanying witnesses to tribunals. Only one interviewee, from a private health and social care provider, said their organisation provided very little support.

‘Interviewer: Would there be any specific support offered to [colleague witnesses]?

No. We’ve had this question before and HR director has said a witness in a hearing should have should attend without assistance… As they are a registered person there may be support systems within the organization that they are registered with.’ (Interview 23).

##### Harmed colleagues and appropriateness of FtP

Some interviewees questioned the appropriateness of FtP referrals when a registrant was alleged to have harmed a colleague rather than a patient or service user. Interviewees were asked about these cases, but allowed interviewees to interpret ‘harm’ for themselves. There was a noticeable difference in the responses from interviewees who interpreted this harm as sexual harassment/assault and those who interpreted harm as bullying. Sexual harassment/assault was always felt to be appropriate for FtP referral. One interviewee reported that an allegation of sexual harm

‘would generally result in the immediate suspension of the person accused and the support [would go] to a person who has been affected by an incident… of sexism, or assault’ (Interview 23).

Where harm was interpreted as bullying however, interviewees stated that an FtP referral was not always required. A further division was noted here – if interviewees assumed that they as the employer had referred a registrant for alleged harm to colleagues due to bullying, they felt referral was a valid mechanism.

The fact that we’ve gone to fitness to practise suggests that some part of it’s been substantiated otherwise it wouldn’t have gone there (Interview 12).

In five interviews, interviewees assumed the allegedly harmed colleague had made a direct FtP referral themselves based upon bullying by a colleague. These interviewees then questioned the validity of the referral, in each case reporting that they felt this kind of alleged harm should be investigated by the employer, and questioned the motivation of employees who made direct referrals based upon bullying.

‘It’s really difficult because sometimes it’s malicious. And sometimes it’s quite hard to differentiate what’s malicious and what’s factual. But we investigate them all objectively, because we have to. But sometimes you do have a niggling because you know relationships. Sometimes when they do it, colleagues do it about other colleagues they often do it anonymously to the [regulator].’ (Interview 9).

#### Public witnesses

##### Understanding of public witnesses’ needs

There was a wide difference in the experiences of public witnesses amongst interviewees, with many stating that they had never encountered a public witness in an FtP case. This was usually in a social care context, whilst interviewees from general hospital or community healthcare context had a range of experience of public referrals.

Interviewees described FtP processes as engendering equal distress amongst public witnesses as amongst registrants. A number of similar issues were discussed by interviewees, namely that the whole system was too formal and that the length of time between a referral and a hearing was harmful to public witnesses.

‘For most patients it’s an entirely alien world’ (Interview 2)

‘some witnesses… are extremely upset. They’re coming into… a very formal process… I’ve had a lot of witnesses surprised… people have said it’s like a court… people are very uncomfortable with the formality often’ (Interview 22).

Compared to knowledge about referred registrants’ and colleague witnesses’ needs, many interviewees had significantly less knowledge about what public witnesses might need. Some expressed surprise at the question, whilst others explained that they had no knowledge of this groups’ needs because of a gap in communication with the regulator. This could either result in them not knowing the identity of a public witness, or where they did know this, not knowing whether or when the person had been called as a witness.

Where interviewees did describe public witnesses’ needs, they focussed on informational rather than emotional needs. Family and friend referrers were perceived to need information about what had happened to their relative/friend, whilst those who alleged harm were seen as needing to be heard by the organisation.

##### Responses to public witnesses’ needs

Some interviewees described uneasiness at the idea it might be their organisation’s role to provide support to public witnesses, assuming the relationship would have irretrievably broken down by the point that an FtP referral was made:

‘That’s a really tricky question because invariably the relationship with the employer and the patient or the family tends to be very poor. It’s never actually occurred to me to reach out to provide support to families in that situation.’ (Interview 3).

Considering the employer’s complaint services, interviewees described providing significant support generally, and talked about specific cases where support had been offered:

‘We always offer local resolution meetings… there’s been a case recently [where] we’ve worked really, really hard to try and work with the advocate and the patient to understand their ongoing concerns and how we can manage those.’ (Interview 6).

However, interviewees talked less about the needs and support offered to public witnesses during FtP. In many cases interviewees said no support was offered by their organisation. Some interviewees said the organisation provided support, but the support they discussed centred around creating or maintaining an ongoing relationship with the patient/service users’ family. Examples that interviewees described included providing medical assistance, carrying out their own investigation, listening to their account, apologising, and offering updates and reassurance. ‘Whatever means of communication they prefer, whether that’s telephone calls and updating them as to the investigation and what’s happening. Or whether that’s letter correspondence and it can include help with regards to welfare and seeking further medical treatment should it be needed.’ (Interview 16).

This is more properly described as an organisational response, rather than support, as it is based upon the organisation’s duties rather than being responsive to identified needs of the public witnesses. Only one organisation described providing both an advocate and practical support for service users:

‘Someone to explain to them who’s going to be there, what questions are going to be asked… how they prefer to be separated from [the staff member] … this is something we’re asking them to do so they need to be compensated, like any of us would be for doing additional stuff.’ (Interview 12).

Interestingly this organisation was one of the few that assumed a care relationship would be continuing whilst a service user was a witness in FtP procedures, which would be typical of those providing care to people with learning disabilities or serious mental illnesses.

Table 4 summarises the differences in provision of organisational response or support for registrants, colleague witnesses and public witnesses.

**Table 4:** Percentage of interviewees describing some form of organisational support offered and to whom

Organisational type	Referred registrants	Colleague witnesses		Public witnesses	
		Unharmed	Harm alleged	Unharmed	Harm alleged
NHS	100%	100%	100%	58%	67%
Private health and/or social care provider	100%	100%	100%	67%	83%
Corporate pharmacy business	100%	100%	100%	67%	67%
Public sector social care	100%	75%	100%	25%	25%
**Total**	100%	96%	100%	56%	64%

## Discussion

Employers were aware of the toll taken on referred employees, exacerbated by the time to case resolution. This could impact relationships within teams as well as trust in those referred. Employers strongly opined that changes to professional regulation were needed to improve the targeting of the most serious cases, and a few wanted the system of FtP to be used differently, or replaced with less adversarial approaches.

All interviewees recognised that their organisations should support employees facing FtP proceedings, and felt the support their organisation provided was appropriate. Unsurprisingly, support resembled that usually provided during a disciplinary investigation; indeed, many regulatory referrals arise from such investigations. However, FtP support might need to extend over years, with implications for the team members’ deployment and registrants’ and colleague witnesses’ practice during that extended period.

Unharmed colleague witnesses who have observed alleged misconduct were offered similar support to referred registrant employees. The extent of preparation for appearing as a tribunal witness for the regulator differed markedly between organisations. All would signpost to the regulators’ websites, although these were regarded as inadequate. The provision of practical and relational support such as accompanying them to a Hearing was more likely to be offered if the interviewee themselves had experience of an FtP tribunal. A further difference came in responses to the allegations if a colleague was harmed. Some felt that bullying, as opposed to sexual harassment, was better dealt with by local investigation only, citing a risk that allegations of bullying could be vexatious.

There was acknowledgement that employees should routinely be made aware of the role of their regulator and FtP processes, but this seldom featured in induction or continuing professional development. Exceptions came again from interviewees who had been professional witnesses in an FtP tribunal, or (more rarely) had been referred themselves. These interviewees were more likely to offer direct support to their employee witnesses, in line with their duty to provide organisational support [20] and to ensure that staff were made aware of FtP in their training. All felt their training resources for staff were insufficient.

In contrast, support offered to the public (service users/patients and their families) was far less than that offered to employees. Of the 25 organisations interviewed, only 14 offered support for all public witnesses, compared to 24 who offered support for all colleague witnesses. What was offered by these 14 organisations was also very limited. In most cases the offer for public witnesses during FtP was lower than that offered during a local complaint process, whilst the support available to registrants and colleague witnesses during FtP processes was based on that offered during a local investigation, but had been further developed in recognition of the specific needs of these groups during FtP. It is possible that employers view an FtP referral as the end of local investigation, and therefore withdraw the support they offer to the public during a local investigation. Alternatively, it could that where a public witness makes the referral themselves, the mismatch between the employer’s view of the situation and the public witness’ view result in a dearth of support.

Some employers were also unable to offer support to the public, as they were not aware of the regulatory referral/complaint until the FtP investigation had proceeded. In some cases, there was reluctance to open up irretrievably broken relationships, especially if there was a possibility of clinical negligence lawsuits, yet these patients/service users sometimes still needed to access services, and had little choice but to remain with this provider – a situation which no organisation had support in place to provide. The only exception to this was one provider (long-term mental health and learning disability services), which offered personal and practical support (including at the tribunal), to rebuild the person’s trust in the provider.

As with registrants and colleague witnesses, current informational resources provided by the regulators were deemed insufficient by interviewees, but no interviewee reported that their organisation provided additional informational resources to compensate for this. Interviewees stated that their organisations typically do not give support to patients/service users to complain to other organisations, such as when the process is led by the regulator, even if they remain under their care, or suffer after-effects of poor care delivered by the health or social care organisation.

The project of which this is a sub study [27] includes both analyses of the information for the public about FtP by the regulators and observations and interviews with public participants in the FtP processes, and will produce free on line resources for the public and professionals about FtP.

## Conclusions

Whilst employing organisations have recognised the need to support registrants who are under FtP investigation, there was a greater variation in the support offered to colleague witnesses. When the interviewee had experience of FtP processes, greater support was likely to be offered to colleague witnesses. Few organisations provided any support for members of public who were witnesses, and the range of support that was available from these few organisations was extremely limited.

This research has implications for policymakers and managers across health and social care organisations in the UK. Table 5 demonstrates the implications arising from the main findings of this research.

**Table 5:** Main findings from and implications of research

Finding	Implications for health services managers
Employers interviewed in this research had a good understanding of the wellbeing and informational needs of registrants who are referred into FtP processes, and those who are called as witnesses for the employer. Employers who had themselves been a witness or a referred by a registrant demonstrated greater understanding of their relational needs.	Where a manager’s role includes responsibility for referred registrants, consideration should be given to ensuring they have informational, wellbeing and relational support, including an experiential understanding of the experiences of referred registrants and witnesses. This might include attending as observers to regulator’s hearings held in public.
Despite responsibilities to respond when patients and service users complain about their employee, few considered they should extend this care to if the patient or service user made a direct FtP referral .	Managers should consider extending support to those who complain to a regulator about their employee.
Overall, employers offered support to those involved in FtP process in any capacity based on the support they would offer during local investigations. Such support may not be sufficient for lengthy FtP processes.	Consider whether the support in place for employer investigations is appropriate to meet the needs of referred registrants, colleague witnesses and public witnesses during often lengthy FtP processes.
Employers identified clear gaps in the resources available from themselves and the regulators to prepare anyone who was called to a regulatory Hearing.	In the absence of sufficient resources emerging from regulators, health services may need to identify alternative resources, or commission their own individualised resources.

## Supplementary information

Supplementary Material 1

## Data Availability

The datasets generated and/or analysed during the current study are not publicly available due to the potential identifiability of individual interviewees but are available from the corresponding author on reasonable request.

## Abbreviations

UK: United Kingdom
FtP: Fitness to Practise
