# Supplementary material for: Employer support for health and social care registered professionals, their patients and service users involved in regulatory fitness to practise regulatory proceedings
Source: BMC Health Serv Res. 2024 Oct 22;24:1268. doi: 10.1186/s12913-024-11646-0 (PMC11494793; doi:10.1186/s12913-024-11646-0)
Supplement: Supplementary file 1 — Supplementary Material 1 [file 12913_2024_11646_MOESM1_ESM.docx]

**WP1.4 EMPLOYERS’ INTERVIEW SCHEDULE**

1. Please describe your job role in (ORGANISATION) (prompts- does this include staff disciplinary investigations, adverse event /patient safety Root Cause Analysis /complaints investigation and reporting?).
2. Please describe your responsibilities for referring concerns about employees to their regulator?
3. Are there others in your organisation who have similar responsibilities for other staff? If so whom, and for which professions?
4. Does (ORGANISATION) keep records of how many staff are referred and the outcomes?
5. Is there any reflection/learning undertaken on the referrals? Can you give me an example of this?
6. What considerations as an employer do you have of the colleagues of the registrant who might have witnessed alleged misconduct?
7. If the colleague had been directly harmed (eg bullied) does the (ORGANISATION) have any duty of care to them and how is this discharged?
8. If the colleague who has been harmed by a registrant is asked to be a witness in a fitness to practise hearing, what support if any is offered by (ORGANISATION) ?
9. What in your view needs to be improved by (ORGANISATION) to make being a witness less distressing for colleagues?
10. Is there anything you think the regulators might do better to make being witness less distressing for colleagues? Please explain.
11. If a patient/service user or their family are asked to be a witness in fitness to practise hearing, what support, if any, is offered by (ORGANISATION)?
12. Would this support be any different if they had been harmed by the registrant’s alleged misconduct? Please explain.
13. What in your view needs to be improved by (ORGANISATION) to make being witness less distressing for patients/service users and families?
14. Is there anything you think the regulators might do better to make being a witness less distressing for patients/service users and families?
15. When considering making new referral of a registrant to FtP, or tracking current cases, there a point of contact or liaison officer of the regulator you can discuss this with?
16. Is there anything else you would like to add?
17. Would you like to have summary of the research you have taken part in today sent you afterwards? If so, may we store your e mail address for this purpose?
